# Supplementary figures and images for: Classifying Interactions in a Synthetic Bacterial Community Is Hindered by Inhibitory Growth Medium
Source: mSystems. 2022 Oct 5;7(5):e00239-22. doi: 10.1128/msystems.00239-22 (PMC9600862; doi:10.1128/msystems.00239-22)

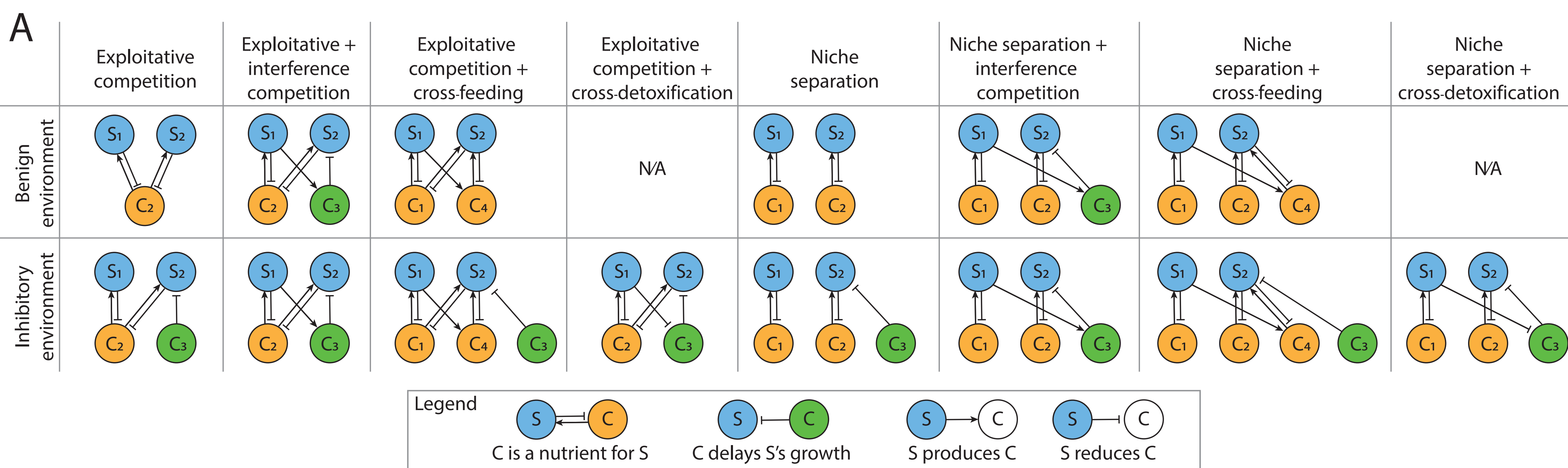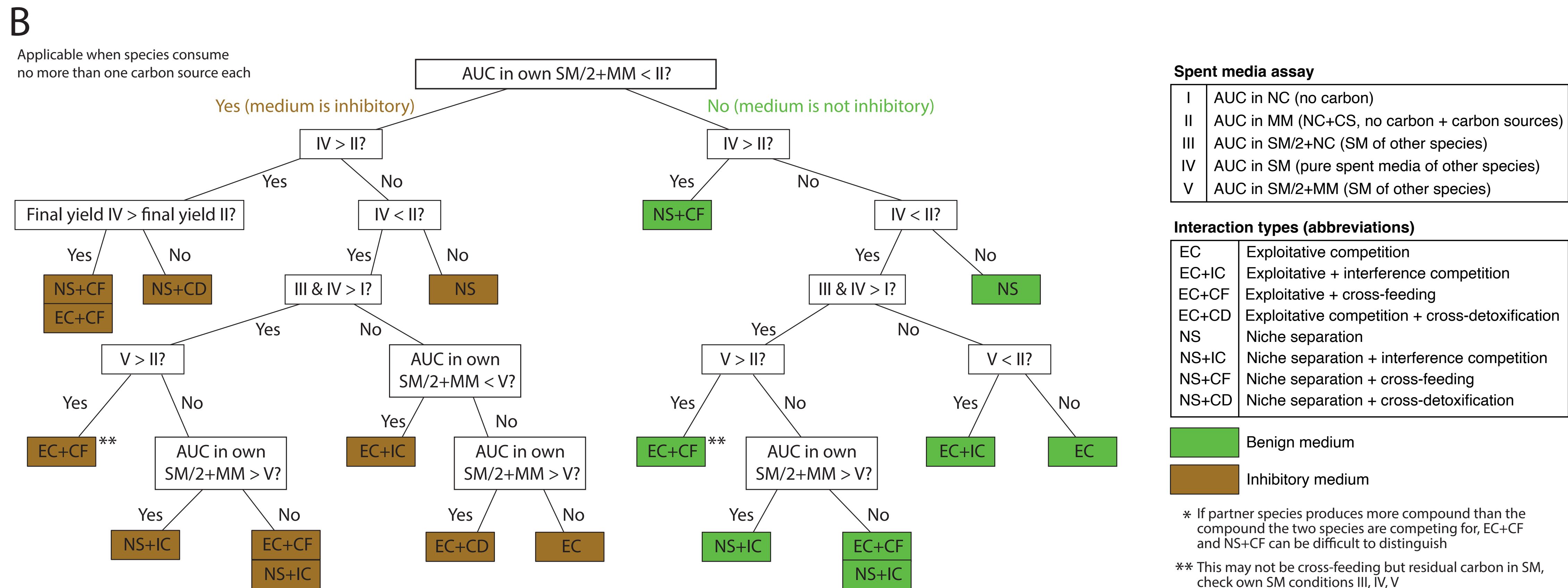

Supplement: FIG S1 [file msystems.00239-22-s0003.pdf]

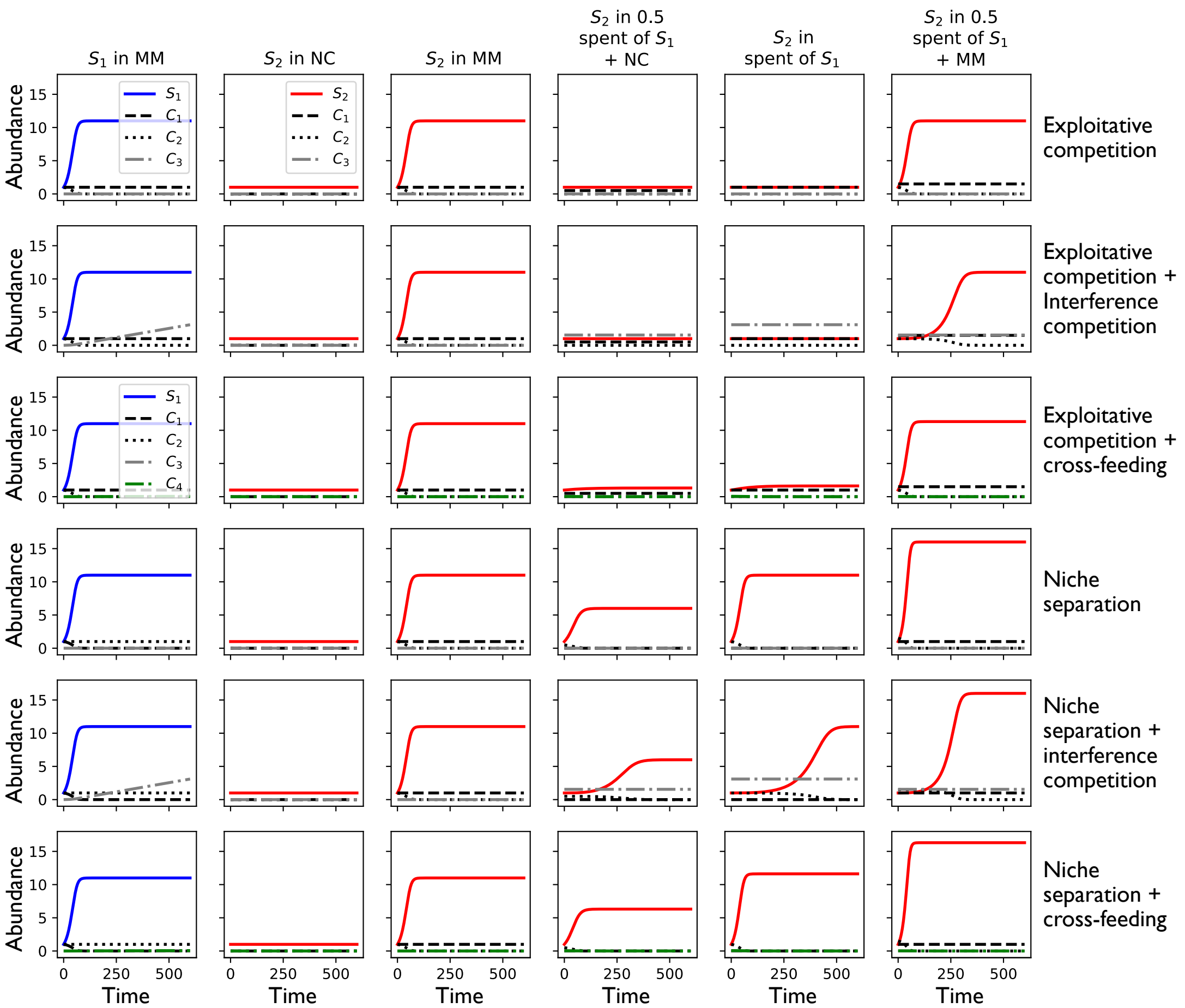

Supplement: FIG S2 [file msystems.00239-22-s0004.pdf]

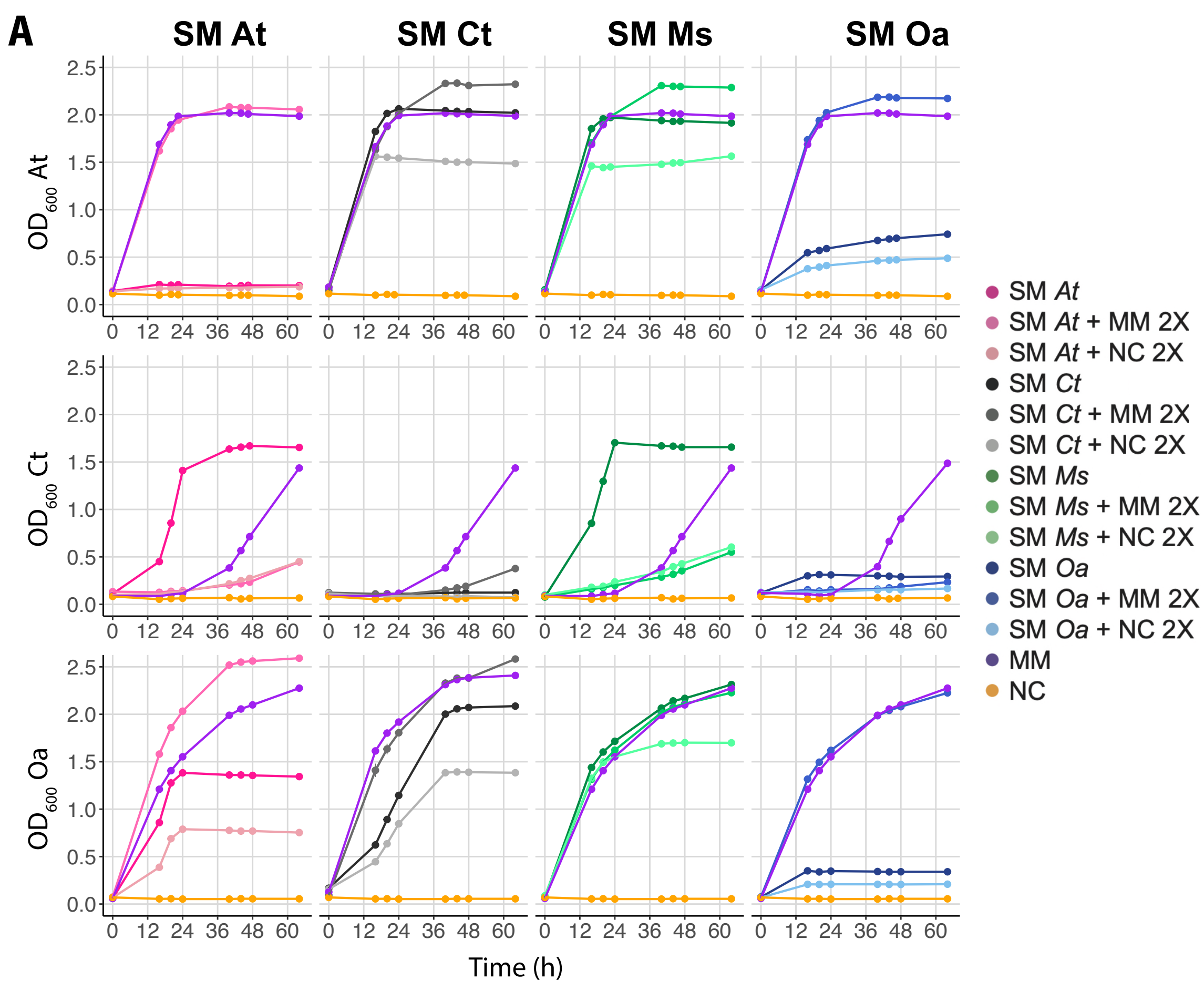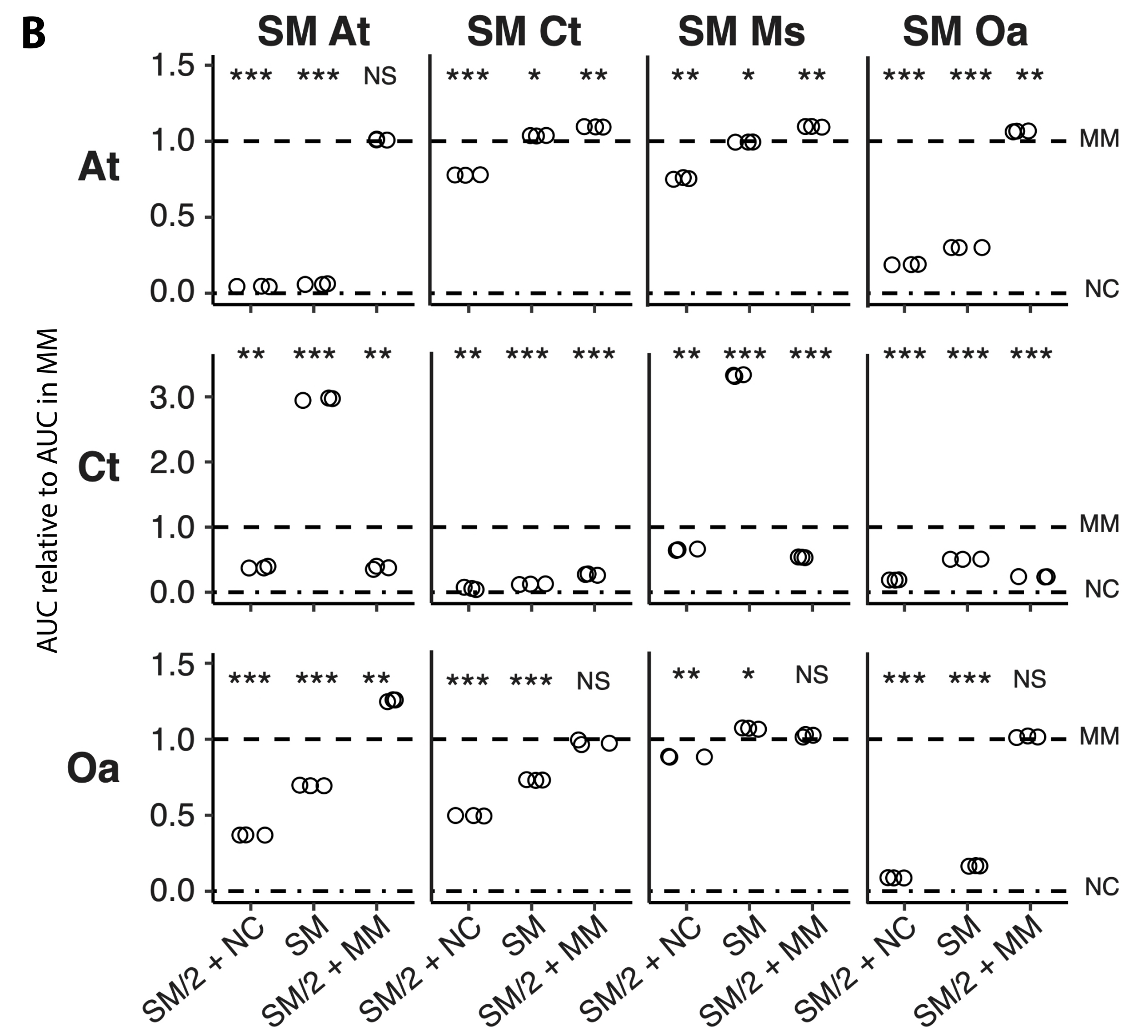

Supplement: FIG S3 [file msystems.00239-22-s0005.pdf]

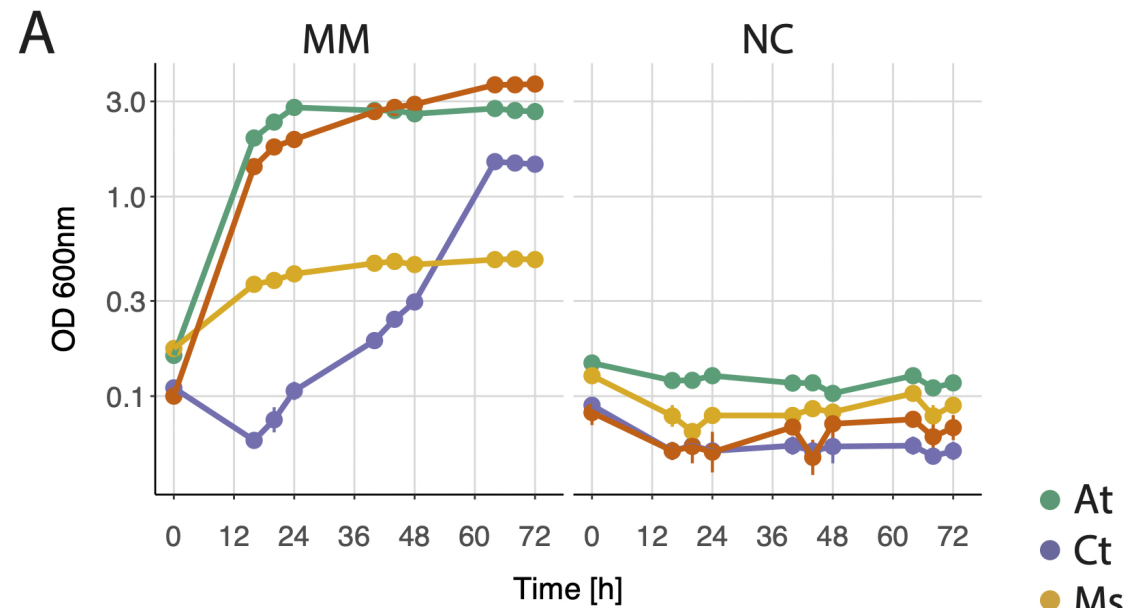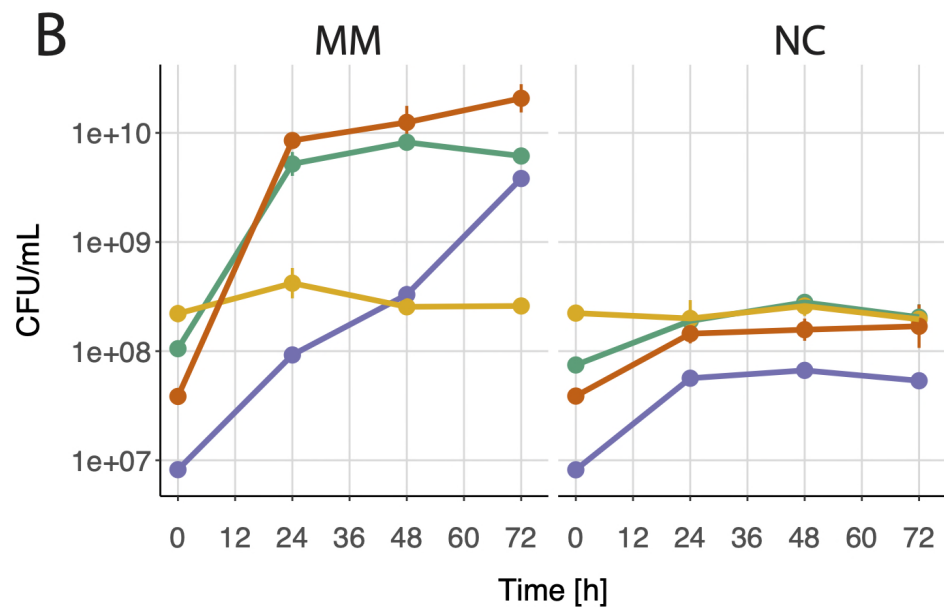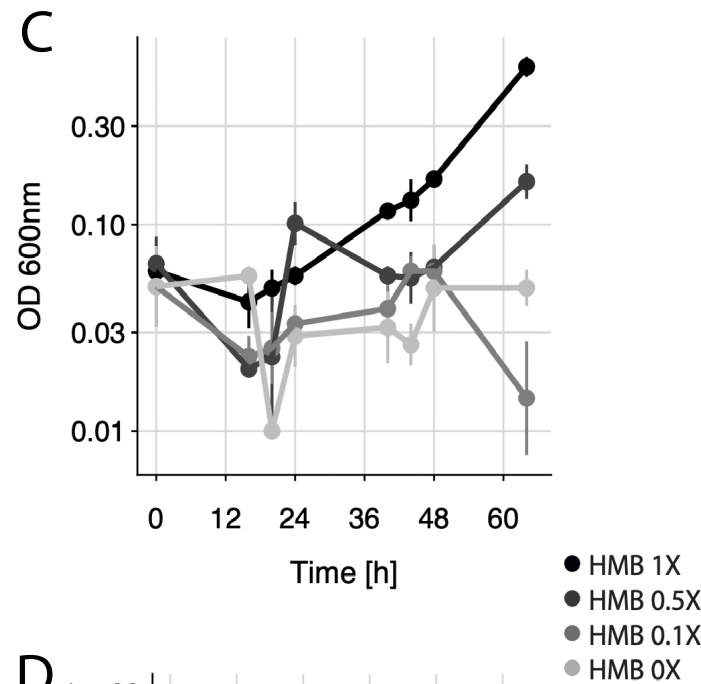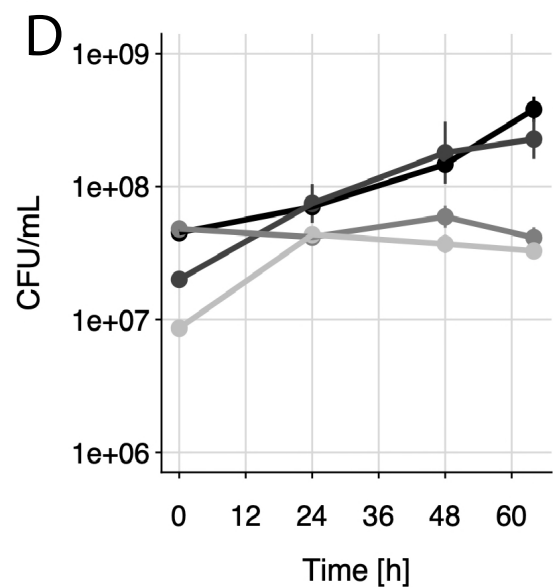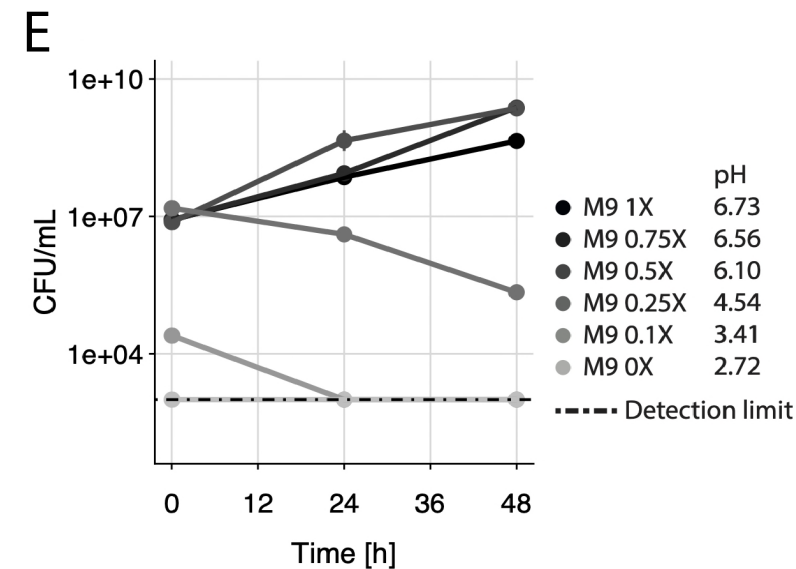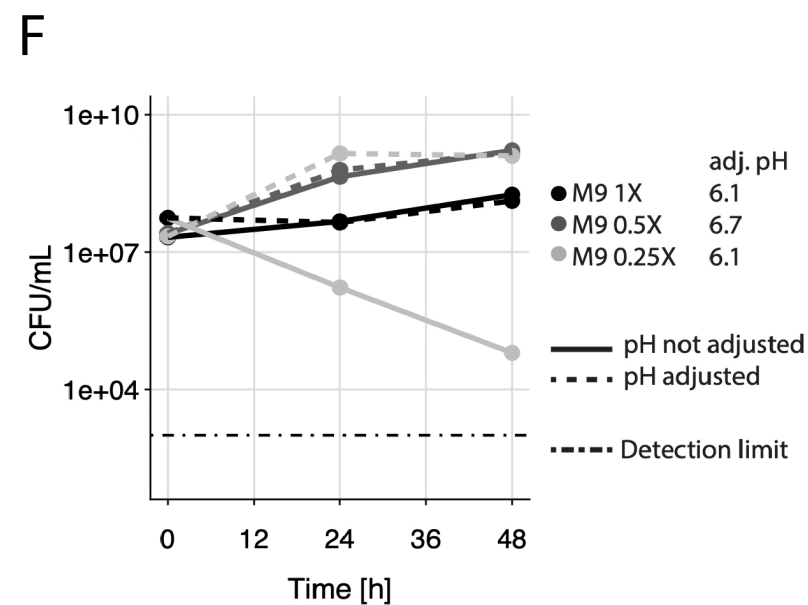

Supplement: FIG S4 [file msystems.00239-22-s0006.pdf]

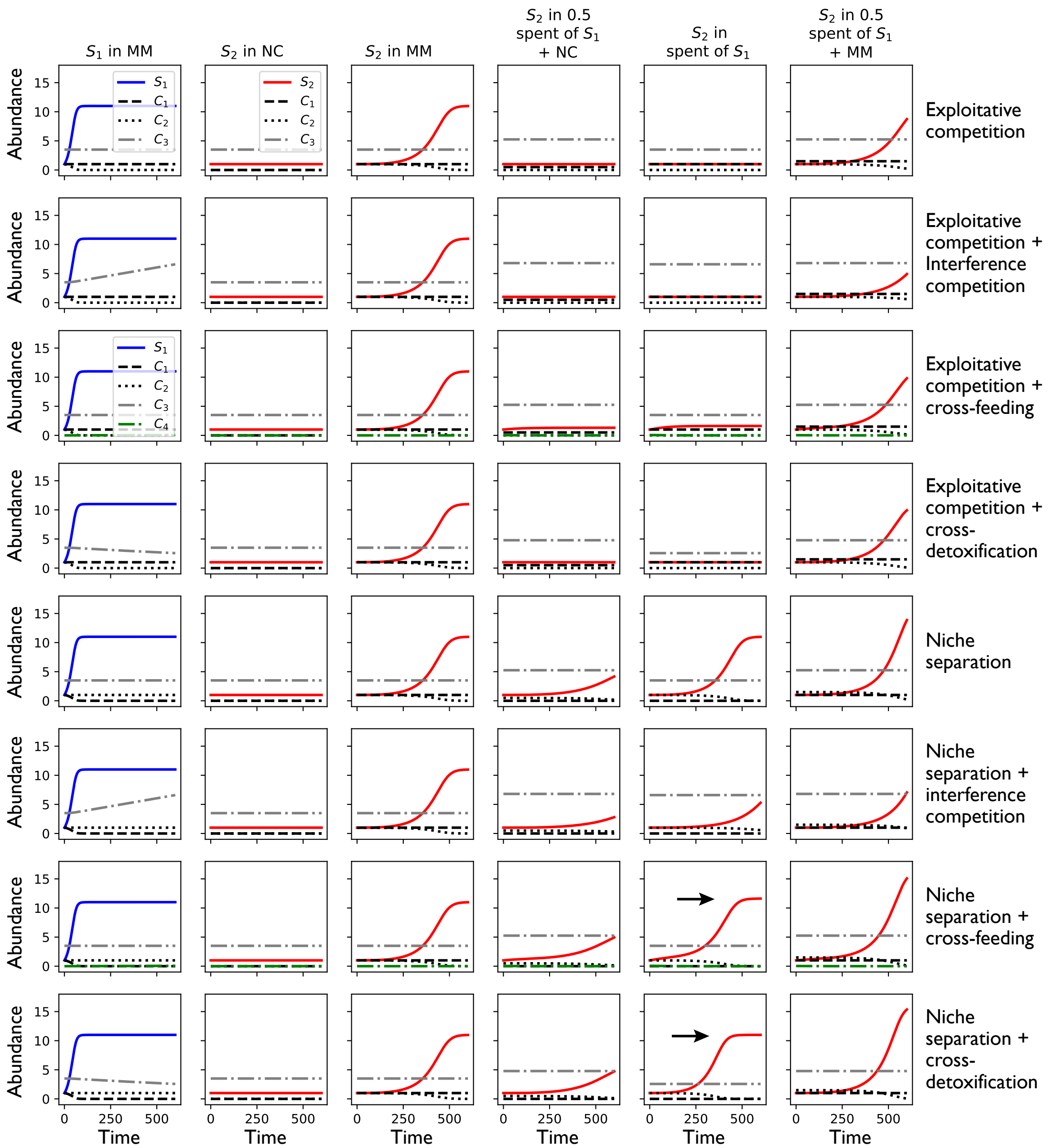

Supplement: FIG S5 [file msystems.00239-22-s0007.pdf]

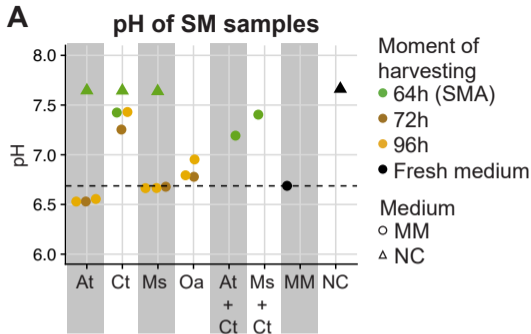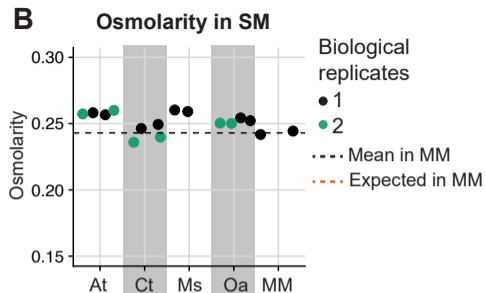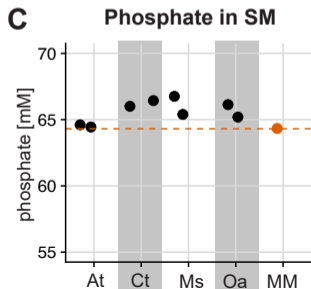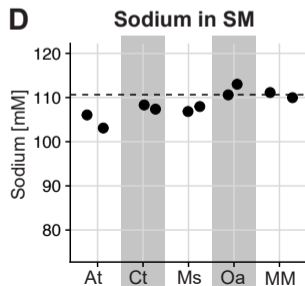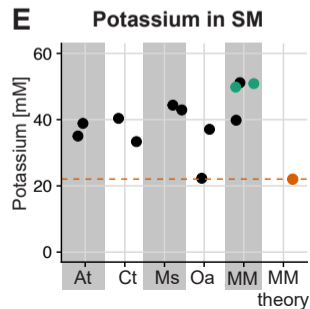

Supplement: FIG S6 [file msystems.00239-22-s0008.pdf]
