# Supplementary material for: Classifying Interactions in a Synthetic Bacterial Community Is Hindered by Inhibitory Growth Medium
Source: mSystems. 2022 Oct 5;7(5):e00239-22. doi: 10.1128/msystems.00239-22 (PMC9600862; doi:10.1128/msystems.00239-22)
Supplement: TEXT S1 [file msystems.00239-22-s0001.pdf]

Supplementary Note S1:

Metabolomics analyses. Metabolite extraction: To extract the metabolites, 400 μL of ice-cold MeOH was added to 100 μL of each medium. The samples were then vortexed for 30 seconds, followed by 15 min centrifugation at 13,000 rpm at 4° C. The resulting supernatant was collected and evaporated to dryness in a vacuum concentrator (LabConco, Missouri, US) and the dried extract was reconstituted in 100 μL of 80 % MeOH prior to LC-MS injection (3).
Data acquisition - LC-HRMS analyses: Media extracts were analyzed by Hydrophilic Interaction Liquid Chromatography coupled to high resolution mass spectrometry (HILIC - HRMS) in both positive and negative ionization modes using a 6550 Quadrupole Time-of-Flight (Q-TOF) system interfaced with 1290 UHPLC system (Agilent Technologies) as previously described (2). In positive mode, the chromatographic separation was carried out in an Acquity BEH Amide, 1.7 μm, 100 mm × 2.1 mm I.D. column (Waters, Massachusetts, US). Mobile phase was composed of A = 20 mM ammonium formate and 0.1 % FA in water and B = 0.1 % formic acid in ACN. The linear gradient elution from 95% B (0-1.5 min) down to 45% B was applied (1.5 min -17 min) and this conditions were held for 2 min. Then initial chromatographic condition were maintained as a post-run during 5 min for column re-equilibration. The flow rate was 400 μL/min, column temperature 25 °C and sample injection volume 2μl. In negative mode, a SeQuant ZIC-pHILIC (100 mm, 2.1 mm I.D. and 5 μm particle size, Merck, Damstadt, Germany) column was used. The mobile phase was composed of A = 20 mM ammonium Acetate and 20 mM NH4OH in water at pH 9.7 and B = 100% ACN. The linear gradient elution from 90% (0-1.5 min) to 50% B (8-11 min) down to 45% B (12-15 min). Finally, the initial chromatographic conditions were established as a post-run during 9 min for column re-equilibration (5). The flow rate was 300 μL/min, column temperature 30 °C and sample injection volume 2μl. Mass spectrometry ESI source conditions were set as follows: dry gas temperature 290 °C and flow 14 L min-1, fragmentor voltage 380 V, sheath gas temperature 350 °C and flow 12 L min-1, nozzle voltage 0 V, and capillary voltage +2000 V in positive mode and -2000 V in negative ionization mode. The instrument was set to acquire over the full m/z range 50-1000 in both modes, with the MS acquisition rate of 2 spectra/s. In addition, AIF (all ion fragmentation) MS/MS analysis were performed on pooled QC samples at a collision energy (CE) of 0, 10 and 30 eV.
Quality control (QC): Pooled QC samples (representative of the entire sample set) were analyzed periodically (every 6 samples) throughout the overall analytical run in order to assess the quality of the data, correct the signal intensity drift (attenuation in most cases, that is inherent to LC-MS technique and MS detector due to sample interaction with the instrument over time) and remove the peaks with poor reproducibility (CV $>$ 30%) (1). In addition, a series of diluted quality controls (dQC) were prepared by dilution with methanol: 100% QC, 50% QC, 25% QC, 12.5% QC and 6.25% QC and analyzed at the beginning and at the end of the sample batch. This QC dilution series served as a linearity filter to remove the features which don't respond linearly or for which the correlation with dilution factor was $<$ 0.75.
Data (pre) processing: Raw LC/MS files were processed using Profinder B.08.00 software (Agilent Technologies) for metabolite identification using an in-house database containing around 600 metabolites. Metabolites were identified based on accurate mass and retention time matching against standards solutions characterized under the same LC-MS conditions and the parameters settings were as follows: Match tolerance masses 10 ppm, Retention time tolerance 0.2 min, height filter 1000 counts, peak spectrum obtained as an average of scants at 10% of the peak. The relative quantification of metabolites was based on EIC (Extracted Ion Chromatogram) areas. The obtained tables (containing peak areas of detected metabolites across all samples) were exported to "R" software `http://cran.r-project.org/` and signal intensity drift correction was done within the LOWESS/Spline normalization program (5) followed by noise filtering (CV (QC features) $>$ 30%).
Metabolite identification: Short listed ions of interest were matched against the in-house created Accurate Mass Retention Time (AMRT) database to confirm the metabolite identities. Putatively identified metabolite features were further subjected to fragmentation (MS/MS data) pattern matching. The metabolite identifications were validated by matching the de-convoluted MS/MS against METLIN standard metabolite database and in-house recorded spectral library acquired on standards (2).
Statistical analyses (univariate): ANOVA one-factor (on $\log 10$ transformed data) was used to test the significance of metabolite changes in different conditions (i.e. different fresh and spent media) with an arbitrary level of significance, p-value $= 0.05$.

Mathematical model. Consider two species that each grow alone in a well-mixed culture medium containing several chemical compounds. The concentration C_j of each compound j can affect the abundance S_i of each species $i \in \{1,2\}$ via feeding or growth retardation. When compound j is a nutrient, its effect on S_i follows the growth function ρ_i that saturates with increasing

DRAFT

compound concentrations (Monod growth) and the concentration of compound j decreases as a function of S_i via the biomass yield $Y_{i,j}$ and growth function ρ_i. Our first version of the model considered only such positive effects of compounds on growth. In our second version of the model (see discussion section of the main text), we augmented our model such that a compound k can possibly have a negative effect on S_i by increasing its lag phase. Based on our experimental observations, the lag phase is increased only for growth on certain compounds j when compound k is present. This is captured by the second term in the growth function ρ_i and modulated by lag factor $l_{i,j,k}$. Finally, compound j can be produced by species i at rate $p_{i,j}$, or passively taken up at rate $u_{i,j}$. This results in the following set of differential equations:

$$\frac{dS_i}{dt} = \sum_j \rho_i(C_j)S_i \tag{1a}$$

$$\rho_i(C_j) = r_{i,j}\frac{C_j}{C_j + K_{i,j}}\frac{t^n}{(\sum_{k\neq j} l_{i,j,k}C_k)^n + t^n} \tag{1b}$$

$$\frac{dC_j}{dt} = \begin{cases} -\frac{1}{Y_i}\rho_i(C_j)S_i & \text{if } C_j \text{ is a nutrient for species } i \\ p_{i,j}S_i & \text{if } C_j \text{ is produced by species } i \\ -u_{i,j}C_jS_i & \text{if } C_j \text{ is passively taken up by species } i \end{cases} \tag{1c}$$

$Y_{i,j}$ is always 0.1, $K_{i,j}$ is always 1.0. The exponent n is 2 and t refers to time. The different interaction types are illustrated in Fig. S1A. Depending on the type of interaction modeled, parameters $r_{i,j}$, $p_{i,j}$ and $l_{i,j}$ can vary as indicated in Table S1 below. Values not listed there (e.g. $r_{2,1}$) are 0 throughout all our simulations. The model was implemented in Python 3.8.2 using the SciPy library v1.7.1. ODEs were solved using scipy.integrate.ode and integrator dopri5, which uses the Runge-Kutta method. Time-courses of 600 time-steps (arbitrary units) were simulated.

The model is largely based on that developed in (4). Here, we have added a lag phase that is a function of the concentration of chemical compounds, and the production of compounds that can be cross-fed or that increase the lag phase.

DRAFT

References

1. Warwick B. Dunn, David Broadhurst, Paul Begley, Eva Zelena, Sue Francis-Mcintyre, Nadine Anderson, Marie Brown, Joshau D. Knowles, Antony Halsall, John N. Haselden, Andrew W. Nicholls, Ian D. Wilson, Douglas B. Kell, and Royston Goodacre. Procedures for large-scale metabolic profiling of serum and plasma using gas chromatography and liquid chromatography coupled to mass spectrometry. *Nature Protocols 2011 6:7*, 6(7):1060–1083, 6 2011.
2. Héctor Gallart-Ayala, Ioana Konz, Florence Mehl, Tony Teav, Aikaterini Oikonomidi, Gwendoline Peyratout, Vera van der Velpen, Julius Popp, and Julijana Ivanisevic. A global HILIC-MS approach to measure polar human cerebrospinal fluid metabolome: Exploring gender-associated variation in a cohort of elderly cognitively healthy subjects. *Analytica Chimica Acta*, 1037:327–337, 12 2018.
3. Julijana Ivanisevic, Zheng Jiang Zhu, Lars Plate, Ralf Tautenhahn, Stephen Chen, Peter J. O'Brien, Caroline H. Johnson, Michael A. Marletta, Gary J. Patti, and Gary Siuzdak. Toward 'Omic scale metabolite profiling: A dual separation-mass spectrometry approach for coverage of lipid and central carbon metabolism. *Analytical Chemistry*, 85(14):6876–6884, 7 2013.
4. Philippe Piccardi, Björn Vessman, and Sara Mitri. Toxicity drives facilitation between 4 bacterial species. *Proceedings of the National Academy of Sciences of the United States of America*, 116(32):15979–15984, 8 2019.
5. Hiroshi Tsugawa, Mitsuhiro Kanazawa, Atsushi Ogiwara, and Masanori Arita. MRMPROBS suite for metabolomics using large-scale MRM assays. *Bioinformatics*, 30(16):2379–2380, 8 2014.
