# Supplementary material for: Classifying Interactions in a Synthetic Bacterial Community Is Hindered by Inhibitory Growth Medium
Source: mSystems. 2022 Oct 5;7(5):e00239-22. doi: 10.1128/msystems.00239-22 (PMC9600862; doi:10.1128/msystems.00239-22)
Supplement: FIG S7 [file msystems.00239-22-s0009.pdf]

A

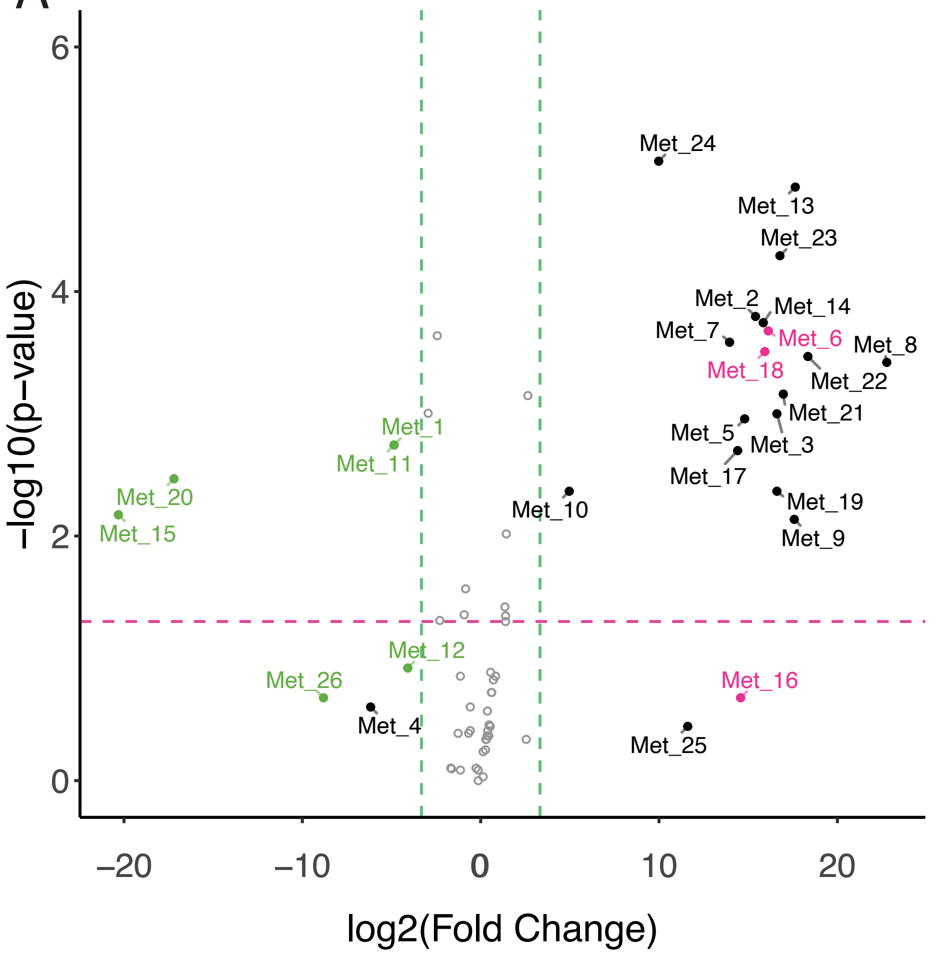

Metabolite	Number
2_5-DIHYDROXYBENZOATE	Met_1
4-GUANIDINOBUTANOATE	Met_2
6-HYDROXYNICOTINATE	Met_3
ALPHA-D-GLUCOSE 2	Met_4
BETA-NICOTINAMIDE_ADENINE_DINUCLEOTIDE	Met_5
CITRULLINE	Met_6
CYTIDINE	Met_7
DEOXYADENOSINE	Met_8
DEOXYCYTIDINE	Met_9
DEOXYGUANOSINE	Met_10
DIHYDROXYBENZOATE_1	Met_11
GALACTOSAMINE	Met_12
GUANOSINE	Met_13
GUANOSINE_MONOPHOSPHATE	Met_14
HYPOXANTHINE	Met_15
MELIBIOSE	Met_16
N-ACETYLGALACTOSAMINE	Met_17
N_N-DIMETHYLARGININE	Met_18
PIPECOLATE	Met_19
PTERIN	Met_20
THYMIDINE -MONOPHOSPHATE	Met_21
URIDINE	Met_22
URIDINE_5 -DIPHOSPHATE	Met_23
URIDINE_DIPHOSPHATE -N-ACETYLGLUCOSAMINE	Met_24
URIDINE_DIPHOSPHATE_GLUCOSE	Met_25
XANTHINE	Met_26

B

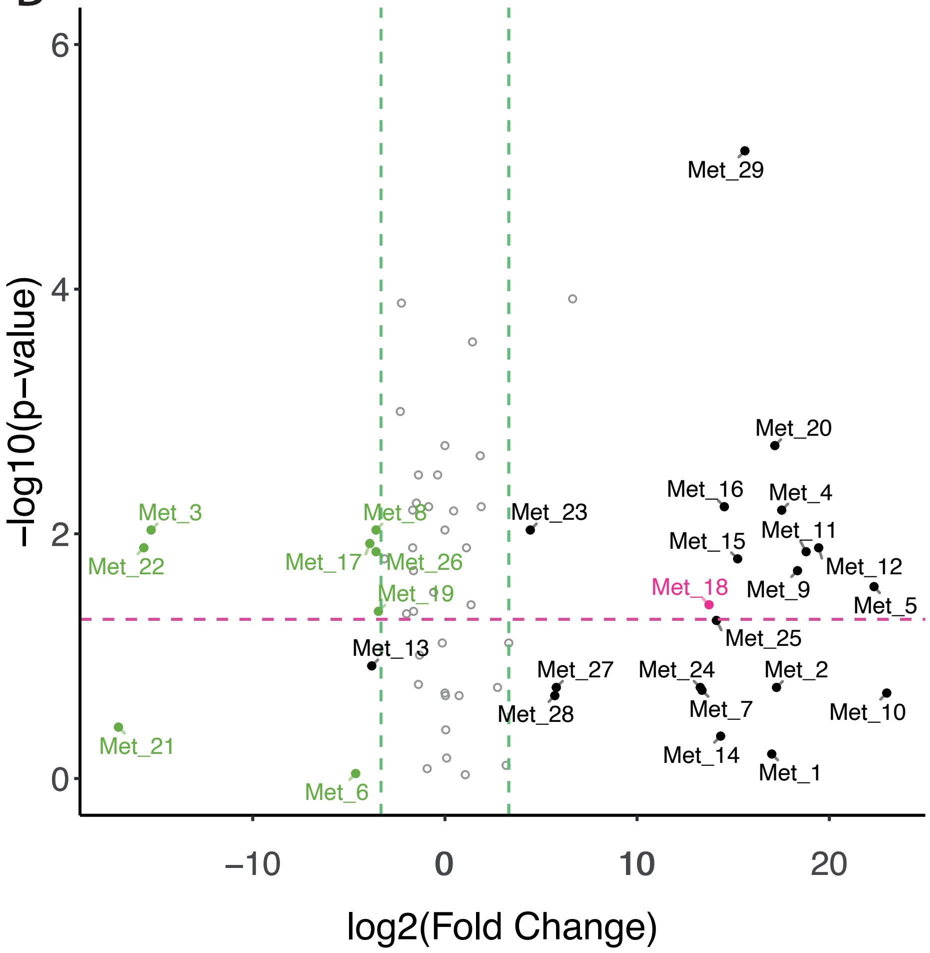

Metabolite	Number
4-ACETAMIDOBUTANOATE	Met_1
4-GUANIDINOBUTANOATE	Met_2
4-HYDROXY -L-PHENYLGLYCINE	Met_3
6-HYDROXYNICOTINATE	Met_4
ADENINE	Met_5
ARGININE	Met_6
BETA-NICOTINAMIDE_ADENINE_DINUCLEOTIDE	Met_7
CIS-4-HYDROXY -D-PROLINE	Met_8
CYTIDINE	Met_9
DEOXYADENOSINE	Met_10
DEOXYCYTIDINE	Met_11
DEOXYGUANOSINE	Met_12
GALACTOSAMINE	Met_13
GUANOSINE_MONOPHOSPHATE	Met_14
N-ACETYLGALACTOSAMINE	Met_15
N-ACETYLPUTRESCINE	Met_16
N-ALPHA-ACETYLLYSINE	Met_17
N_N-DIMETHYLARGININE	Met_18
OXOGLUTARATE	Met_19
PIPECOLATE	Met_20
PROLINE	Met_21
PTERIN	Met_22
PYRIDOXAMINE	Met_23
S-ADENOSYLHOMOCYSTEINE	Met_24
S-ADENOSYLMETHIONINE	Met_25
TRANS -4-HYDROXY -L-PROLINE	Met_26
URACIL	Met_27
URIDINE	Met_28
URIDINE_5 -DIPHOSPHATE	Met_29
