# Supplementary material for: Classifying Interactions in a Synthetic Bacterial Community Is Hindered by Inhibitory Growth Medium
Source: mSystems. 2022 Oct 5;7(5):e00239-22. doi: 10.1128/msystems.00239-22 (PMC9600862; doi:10.1128/msystems.00239-22)

Interaction types (abbreviations)

Exploitative competition	EC
Exploitative + interference competition	EC+IC
Exploitative + cross-feeding	EC+CF
Exploitative competition + cross-detoxification	EC+CD
Niche separation	NS
Niche separation + interference competition	NS+IC
Niche separation + cross-feeding	NS+CF
Niche separation + cross-detoxification	NS+CD

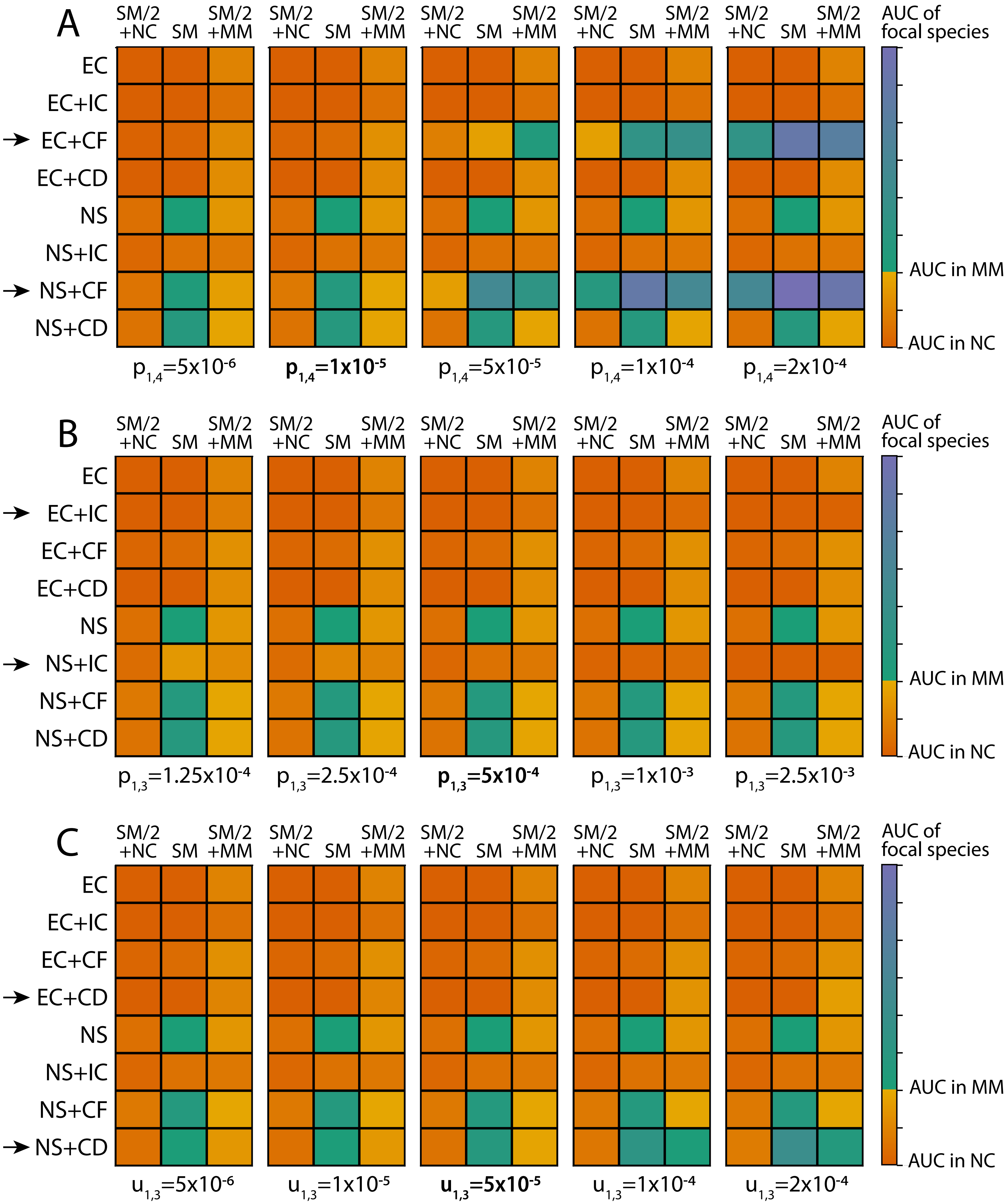

Supplement: FIG S8 [file msystems.00239-22-s0010.pdf]
