# Supplementary material for: Classifying Interactions in a Synthetic Bacterial Community Is Hindered by Inhibitory Growth Medium
Source: mSystems. 2022 Oct 5;7(5):e00239-22. doi: 10.1128/msystems.00239-22 (PMC9600862; doi:10.1128/msystems.00239-22)
Supplement: TABLE S1 [file msystems.00239-22-s0002.pdf]

	EC	EC+IC	EC+CF	EC+CD	NS	NS+IC	NS+CF	NC+CD
$r_{1,1}$	0	0	0	0	0.1	0.1	0.1	0.1
$r_{1,2}$	0.1	0.1	0.1	0.1	0	0	0	0
$r_{2,2}$	0.1	0.1	0.1	0.1	0.1	0.1	0.1	0.1
$r_{2,4}$	0	0	0.1	0	0	0	0.1	0
$p_{1,3}$	0	0.0005	0	0	0	0.005	0	0
$p_{1,4}$	0	0	10^{-5}	0	0	0	10^{-5}	0
$l_{2,2,3}$	200	200	200	200	200	200	200	200
$u_{1,3}$	0	0	0	5×10^{-5}	0	0	0	5×10^{-5}
$S_1(t=0)$	1.0	1.0	1.0	1.0	1.0	1.0	1.0	1.0
$S_2(t=0)$	1.0	1.0	1.0	1.0	1.0	1.0	1.0	1.0
$C_1(t=0)$	1.0	1.0	1.0	1.0	1.0	1.0	1.0	1.0
$C_2(t=0)$	1.0	1.0	1.0	1.0	1.0	1.0	1.0	1.0
$C_3(t=0)$	0 or 3.5	0 or 3.5	0 or 3.5	0 or 3.5	0 or 3.5	0 or 3.5	0 or 3.5	0 or 3.5
$C_4(t=0)$	0	0	0	0	0	0	0	0

Table S1

DRAFT
